# A case-control study coupling with meta-analysis elaborates decisive association between *IGF-1* rs35767 and osteoporosis in Asian postmenopausal females

**DOI:** 10.18632/aging.204464

**Published:** 2023-01-03

**Authors:** Sui-Lung Su, Yung-Hsun Huang, Yu-Hsuan Chen, Pi-Shao Ko, Wen Su, Chih-Chien Wang, Meng-Chang Lee

**Affiliations:** 1 School of Public Health, National Defense Medical Center, Taipei, Taiwan, R.O.C.; 2 Graduate Institute of Life Sciences, National Defense Medical Center, Taipei, Taiwan, R.O.C.; 3 Graduate Institute of Aerospace and Undersea Medicine, National Defense Medical Center, Taipei, Taiwan, R.O.C.; 4 Department of Orthopedics, Tri-Service General Hospital and National Defense Medical Center, Taipei, Taiwan, R.O.C.

**Keywords:** osteoporosis, postmenopausal, insulin-like growth factor-1, single nucleotide polymorphism, meta-analysis

## Abstract

Background: Osteoporosis (OP) is prevalent in postmenopausal women. Several studies investigated the association between *IGF-1* polymorphisms and OP among postmenopausal females with conflicting outcomes.

Objective: To investigate whether the *IGF-1* (rs35767, rs2288377, rs5742612) were associated with OP in postmenopausal females.

Methods: In case-control study, 95 OP cases and 222 healthy controls were recruited between March 2015 and July 2019. OP was diagnosed based on WHO criteria for diagnosis of OP as T score of bone mineral density (BMD) ≤−2.5; normal, as T score of BMD ≥−1. *IGF-1* SNPs were genotyped by iPLEX Gold SNP genotyping. To be solid, related studies from PubMed, Embase, Cochrane, Web of science, and previous meta-analysis up until November 2020, along with our case-control study, were incorporated into a meta-analysis with criteria of significance using odds ratios (ORs) with corresponding 95% confidence intervals (CI) to evaluate risk factor of SNPs on OP. TSA was used to estimate the sample sizes required to achieve a conclusion.

Results: In dominant model of our case-control study, we found nonsignificant association of rs35767 [Adj-OR: 0.95 (95% CI: 0.56-1.60)], rs2288377 [Adj-OR: 1.15 (95% CI: 0.67-1.97)], and rs5742612 [Adj-OR: 1.07 (95% CI: 0.62-1.83)] with OP in postmenopausal females. However, integration of our case-control study and 3 published studies, rs35767 [OR: 1.24 (95% CI: 1.05-1.47)] showed a conclusively risk association with OP in postmenopausal females judged by TSA with 2267 Asians.

Conclusions: This study demonstrates a crucial sample to conclude that *IGF-1* rs35767 is significantly associated with OP in postmenopausal women.

## INTRODUCTION

OP is an important public health problem. Accordingly, OP affects more than 200 million people globally [1]. The pathogenesis of OP is the dysregulation of bone remodelling between osteoclasts and osteoblasts [2], resulting in bone mass reduction and bone tissues deterioration, thereby increasing the risk of bone fracture [3]. There are many risk factors of OP, such as age, sex, lifestyle, and genes [4, 5]. The heritability of OP is 60%–80% [6]. Studies report that network of *IGF-1, COL2A1, DDR2, SOX9,* and *PTH* involve in osteogenesis [7]. *Insulin-like growth factor-1* (*IGF-1*), a growth-promoting cytokine, mediates metabolism, development, and growth [8] and has extremely important effects on bone growth and mineralization together with growth hormone (GH) [9, 10].

Research has unveiled that lower serum concentrations of *IGF-1* significantly associated with the occurrence of osteoporotic fractures in postmenopausal women [11]. In 2002, Lee et al. firstly proposed the relationship of *IGF-1* gene polymorphisms to the bone mineral density (BMD) among postmenopausal women [12]. Sequentially, Rivadeneira et al. reported that the promoter of *IGF-1* with the microsatellite repeat polymorphism was associated with the BMD level and bone loss in postmenopausal women in a large- scale study [13].

Seven studies examine the correlation between *IGF-1* and OP [14–20], of which four studies show that rs35767 is associated with OP in postmenopausal women [14–17]. The results of 2 meta-analysis on *IGF-1* and OP show that rs35767 is associated with OP in postmenopausal women [21, 22]. Besides, other studies also show that *IGF-1*, rs2288377 and rs5742612, are risk factors to OP through regulation of gene expression [20, 23]. Furthermore, three models, the allele, recessive, and dominant inheritance models, were used to evaluate *IGF-1's* predictability for OP, and the dominant model maybe the actual genetic pattern for IGF-1 and OP [22].

Although two meta-analyses have been published, conclusion of the association between *IGF-1* and OP is pending, probably due to limited studies on postmenopausal women [21, 22]. The trial sequential analysis (TSA) provides methodology to assess the creditability of SNP and diseases based on cumulative samples [24]. We designed a case-control study to confirm the relationship of *IGF-1* rs35767, rs2288377, and rs5742612 polymorphisms to OP in Taiwan. To substantialize, we also performed a combination of our case-control study and meta-analysis to conclude the association with *IGF-1* SNPs and OP based on computation of cumulative samples through TSA.

## MATERIALS AND METHODS

### Case-control study

### Subjects

Subjects were collected at the Health Management Center of TSGH in a Taipei check-up program from March 2015 to August 2019. The exclusion criteria included: (1) Total hip replacement, (2) Vertebroplasty, (3) Unsuccessful gene sequencing (Supplementary Figure 1). Demographic data contain age, body mass index (BMI), smoking status, and alcohol consumption status. Finally, 317 older adults were recruited in this research.

OP was diagnosed based on WHO criteria for diagnosis of OP as follows [25]: The case group consists of subjects with a lumbar vertebrae BMD T score of ≤ −2.5 (n = 95), and the control group consists of subjects with a lumbar vertebrae BMD T score ≥ −1 (n = 222).

### Measurement of BMD

BMD was measured based on the density of the lumbar spine (L1-L4) using the Dual energy X-ray absorptiometry (DXA) during the health examinations at TSGH by using Prodigy Series X-Ray Tube Housing Assembly (GE Medical Systems Lunar, Madison, WI, USA) for all the participants [26].

### Genomic DNA extraction and genotyping

Intravenous blood samples (5 mL) from every participant were collected by medical technologists. Genomic DNA of peripheral blood samples was extracted according to standard procedures for proteinase K (Invitrogen, Carlsbad, CA, USA) digestion and the phenol/chloroform method. The polymorphisms of *IGF-1* (rs35767, rs2288377, and rs5742612) were determined using iPLEX Gold SNP genotyping. We adopted inter- and intra-replication validation to evaluate the quality of genotyping experiment, including at least 10% of the samples.

### Statistical analysis

Continuous variables were shown as mean ± standard deviation and tested using Student’s t-test. We used χ2 test to examine whether there were differences in genotype and allele frequencies between OP patients and healthy controls. To identify the risk of OP, we used logistic regression to estimate odd ratios (ORs) and 95% confidence intervals (CIs). Based on previous studies, the dominant model is suggested to be the most suitable model for examining the relationship of the IGF-1 gene to OP [22]. Therefore, in this study, dominant model was used to determine the risk of genetic polymorphisms to OP. The significance was set as a p-value of <0.05. Statistical analyses were performed in R 3.4.4.

### Meta-analysis

### Search methods and criteria of included studies

The PRISMA checklist and Genetic Association Studies Checklist of meta-analysis, including the PROSPERO International Prospective Register of Ongoing Systematic Reviews registration number, CRD42022372332, shown in Supplementary Table 1 [27]. We used related terms of “*IGF-1*” and “OP” to screen the PubMed, EMBASE, Cochrane, and Web of science for articles published up to 31 November 2020 as listed in Supplementary Table 2. Furthermore, articles included in the meta-analysis research were manually checked to avert the omission of vital papers. The criteria of inclusion were listed as following: (1) case-control studies (2) diagnose of OP based on radiological examination (3) genotypes of distribution of *IGF-1* in detail. Types of research were discarded from this analysis: (1) case report, comments, reviews, or animal studies (2) Research on non-postmenopausal women.

### Data extraction

Data quality was assessed by two authors (Sui-Lung Su and Meng-Chang Lee) independently. We recorded the information of each article as following: (1) surname of the first author; (2) year of publication; (3) country where the research was conducted; (4) sample sizes of cases and control subjects; (5) diagnostic criteria of OP; (6) distribution of genotyping in cases and control subjects; (7) Hardy–Weinberg Equilibrium (HWE) test results of control participants. All chosen articles were evaluated using the Newcastle–Ottawa Scale, and all criteria of scores were set as ≥7 points.

### Statistical analysis

We used ORs with 95% CIs to assess the relationship of IGF-1 polymorphisms to the risk of OP in the meta-analysis. The estimation of I^2^ from the DerSimonian–Laird method was adopted to evaluate heterogeneity, and more than 50% of I^2^ exhibited a moderate-to-high heterogeneity [28]. We used the random-effects model to get the summary results. Dominant models were used to calculate the association between genetic polymorphism and the risk of OP. We used Egger’s regression and a funnel plot to examine the symmetry of pooled results [29]. A p-value of <0.05 was set as significant criterion. We used the “meta” packages of R 3.4.4 to perform statistical analyses.

For TSA parameter setting, type I error was set as 0.05, heterogeneity was set as 0%, power was set as 80%, and OR was referenced from previous studies and set as 1.3.

## RESULTS

### Case-control study

Finally, 317 participants were recruited in our case-control study. The demographic data of OP cases and controls is displayed in Table 1. In the control group, there are 222 subjects with a mean age of 71.67 ± 6.19 years. In the case group, there are 95 subjects with a mean age of 71.95 ± 6.76 years. Cases of BMI, waist circumference and hip circumference are significantly lower than those of controls (p < 0.001).

**Table 1:** Distribution of demographic characteristics in the case and control groups.

**Variables**	**Control (N = 222)**	**Case (N = 95)**	**p-value**
Age (Mean ± SD)	71.67 ± 6.19	71.95 ± 6.76	0.702
BMI	25.06 ± 3.72	22.07 ± 3.10	<0.001*
Waist circumference	81.74 ± 9.17	76.01 ± 8.91	<0.001*
Hips circumference	97.83 ± 7.94	92.60 ± 5.67	<0.001*
Smoking			0.637
No	219 (98.6%)	93 (97.9%)	
Current or former	3 (1.4%)	2 (2.1%)	
Drinking			0.310
No	216 (97.3%)	94 (98.9%)	
Current or former	6 (2.7%)	1 (1.1%)	
BMD			
L1	−0.44 ± 1.08	−2.87 ± 0.87	<0.001*
L2	−0.25 ± 1.09	−2.94 ± 0.79	<0.001*
L3	0.09 ± 1.19	−2.99 ± 0.86	<0.001*
L4	0.45 ± 1.54	−2.87 ± 1.17	<0.001*

BMD, bone mineral density.

Control, BMD T score>-1; Case, BMD T score<-2.5; *, p-value< 0.05.

The density of the lumbar spine ranges from L1 to L4.

Results of dominant model show none association between *IGF-1* and OP (Table 2). In univariable analysis, we found that those with CT+TT genotyping may not have higher risk of OP when compared to those with CC genotyping in the rs35767 [Crude-OR: 0.96 (95% CI: 0.60–1.54)]; similarly, the result is comparable after adjustment with age, BMI, smoking, drinking [Adj-OR: 0.95 (95% CI: 0.56–1.60)]. Likewise, there are none association in the rs2288377 [AT+TT vs AA; Adj-OR: 1.15 (95% CI: 0.67–1.97)] and rs5742612 [TC+CC vs TT; Adj-OR: 1.07 (95% CI: 0.62–1.83)] with OP under adjustment with age, BMI, smoking, and drinking. To substantialize, we incorporated samples from the case-control study into the meta-analysis and conducted TSA in sequential analysis.

**Table 2:** Distribution of *IGF-1* genotypes in case and control groups.

	**Control N=222**	**Case N=95**	**Crude-OR (95%CI)**	**p-value**	**Adjust-OR (95%CI)**	**p-value**
rs35767				0.835		0.381
CC	95(42.8%)	39(41.1%)	1		1	
CT	104(46.8%)	44(46.3%)	1.03 (0.62 - 1.72)	0.908	0.91 (0.51 - 1.62)	0.753
TT	23(10.4%)	12(12.6%)	1.27 (0.58 - 2.80)	0.553	1.70 (0.70 - 4.15)	0.24
Dominant				0.876		0.841
CC	95(42.8%)	39(41.1%)	1		1	
CT+TT	127(57.2%)	56(58.9%)	0.96 (0.60 - 1.54)		0.95 (0.56 - 1.60)	
rs2288377				0.843		0.845
AA	114(51.4%)	46(48.4%)	1		1	
AT	88(39.6%)	41(43.2%)	1.15 (0.70 - 1.91)	0.576	1.12 (0.64 - 1.98)	0.689
TT	20(9.0%)	8(8.4%)	0.99 (0.41 - 2.41)	0.985	1.29 (0.48 - 3.48)	0.613
Dominant				0.633		0.610
AA	114(51.4%)	46(48.4%)	1		1	
AT+TT	108(48.6%)	49(51.6%)	1.16 (0.73−1.83)		1.15 (0.67−1.97)	
rs5742612				0.757		0.960
TT	113(50.9%)	47(49.5%)	1		1	
TC	88(39.6%)	41(43.2%)	1.12 (0.68 - 1.85)	0.658	1.08 (0.62 - 1.91)	0.780
CC	21(9.5%)	7(7.3%)	0.80 (0.32 - 2.01)	0.637	1.00 (0.36 - 2.77)	0.996
Dominant				0.783		0.806
TT	113(50.9%)	47(49.5%)	1		1	
TC+CC	109(49.1%)	48(50.5%)	1.08 (0.69−1.70)		1.07 (0.62 - 1.83)	

rs35767 Taiwan biobank MAF: 35%, 1000 Genomes MAF: 30%, HEW: 0.782.

rs2288377 Taiwan biobank MAF: 29%, 1000 Genomes MAF: 10%, HEW: 0.879.

rs5742612 Taiwan biobank MAF: 29%, 1000 Genomes MAF: 11%, HEW: 0.816.

Control: BMD T score>-1, Case: BMD T score<-2.5.

Adj-OR Age, BMI, Smoking, Drinking.

### Meta-analysis

**Figure 1:**
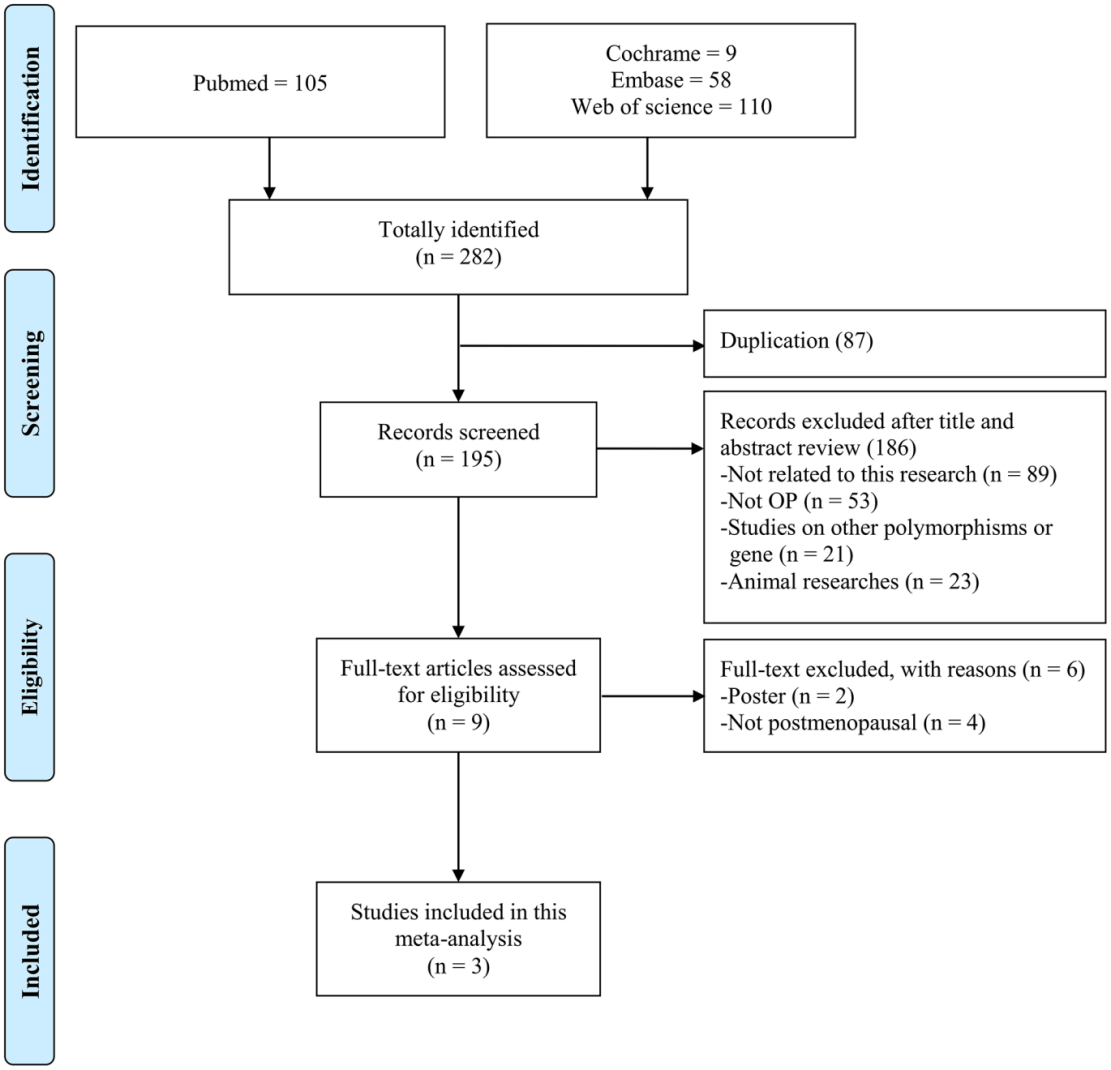
Flow diagram of the identification process for eligible studies.

Flowchart is shown in Figure 1. A total of 282 papers were in this meta-analysis, including 105 papers from PubMed, 58 papers from Embase, 9 papers from Cochrane, and 110 papers from Web of Science. Criteria of exclusion contained repetition, 87 papers; unrelated-genes or -OP topics, 74 papers; animal studies, 23 papers; posters, 2 papers; or non-postmenopausal women, 4 papers. Finally, there were 3 papers in this meta-analysis (Supplementary Table 3) [14–16]. Assessment of methodological quality was evaluated according to Newcastle–Ottawa Scale, and all criteria of scores were set as ≥7 points (Supplementary Table 4).

**Figure 2:**
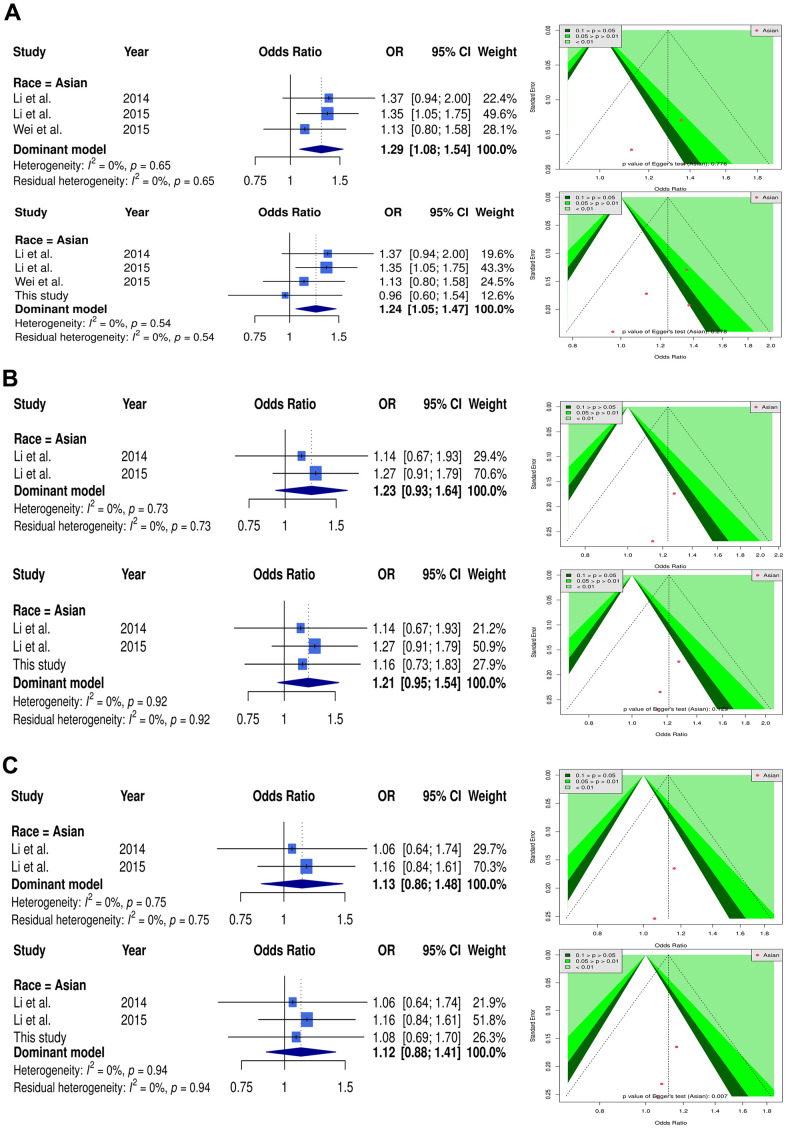
**Forest plot and funnel plot of the association between *IGF-1* and OP using dominant model.** Selected results from the meta-analysis of *IGF-1* and OP. (**A**) The left subplot is a forest plot based on rs35767 dominant model assumption (CT+TT vs. CC). The funnel plot obtained with the dominant models are presented at the right. (**B**) The left subplot is a forest plot based on rs2288377 dominant model assumption (AT+TT vs AA). The funnel plot obtained with the dominant models are presented at the right. (**C**) The left subplot is a forest plot based on rs5742612 dominant model assumption (TC+CC vs. TT). The funnel plot obtained with the dominant models are presented at the right.

Forest plot presents rs35767 in dominant model of meta-analysis and merged case-control study, showing [OR: 1.29 (95% CI: 1.08–1.54)] and [OR: 1.24 (95% CI: 1.05–1.47)], respectively. We adopted a funnel plot to examine the publication bias. None significant result of asymmetry was observed (p = 0.261) (Figure 2A). We also evaluated the meta-analysis results for rs2288377 and rs5742612, but there was no significant difference in the results (Figure 2B, 2C).

### TSA estimation

**Figure 3:**
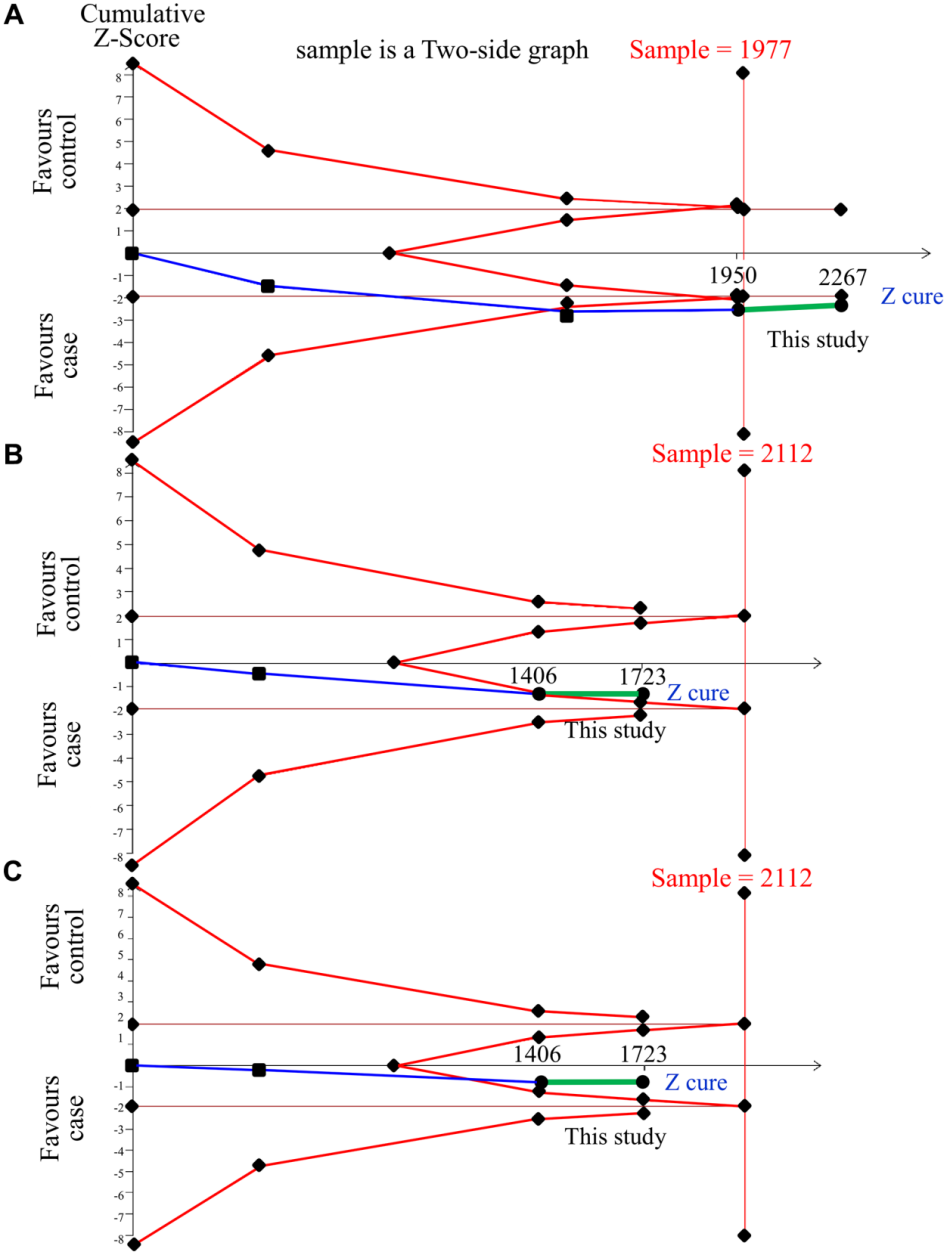
**Trial sequential analysis of *IGF-1* and OP in Asian samples.** We performed a TSA using a dominant model assumption, but replaced the allele count with the sample size (divided by 2). (**A**) rs35767 detailed settings: Significance level = 0.05; Power = 0.8; least extreme OR to be detected = 1.3; MAF=0.35; I^2^ (heterogeneity) = 0%. (**B**) rs2288377 detailed settings: Significance level = 0.05; Power = 0.8; least extreme OR to be detected = 1.3; MAF=0.29; I^2^ (heterogeneity) = 0%. (**C**) rs5742612 detailed settings: Significance level = 0.05; Power = 0.8; least extreme OR to be detected = 1.3; MAF = 0.29; I^2^ (heterogeneity) = 0%.

Figure 3 shows the TSA results to judge the association of *IGF-1* with OP in postmenopausal women. In Asian postmenopausal females, the cumulative sample size, 1950, in rs35767 of meta-analysis was not adequate to achieve a conclusion. However, a joint of case-control research (317 samples) satisfied the minimum sample sizes (1977), criteria of effectiveness, to conclude the risky association between rs35767 and OP. This meta-analysis shows that this study is a crucial sample. The result of TSA in rs2288377 showed that the cumulative sample size was insufficient to reach a conclusion even merging case-control study. The TSA results of rs5742612 is similar to that of rs2288377.

## DISCUSSION

We reveal a conclusive risk association in rs35767 with OP in postmenopausal females judged by TSA with 2,267 Asians in a combination of 3 published studies and our case-control study. However, rs2288377 and rs5742612 show no association with OP but it needs more sample sizes to evaluate the relationship.

However, previous results reported rs2288377 gene polymorphism is significantly associated with OP, while rs35767 and rs5742612 are not significantly correlated with OP [18–20]. Previous meta-analysis studies ignored the estimation of sample size, a crucial key to association study [30], prone to lead to paradoxical conclusion [21, 22]. In this study, we use TSA model to evaluate effectiveness of the meta-analysis through the estimation of sample size. The relationship of the rs35767 to OP is confirmed by TSA with 2267 Asians in a combination of 3 published studies and our case-control study. Bereft of support of sufficient sample sizes in rs2288377 and rs5742612, it needs more research to evaluate the relationship.

The case-control study results found that *IGF-1* polymorphism is somewhat divergent with previous studies [14, 15]. We speculate that the reason probably is much older in our case-control study. Our study population was aged more than 70 years old, while participants in 2 previous papers their age range was 50–68 years [14, 15]. Studies show that serum IGF-1 protein of elderly subjects aged 70 years and above decreases by nearly 60% [31, 32]. IGF-1 mediates bone remodeling and induces the differentiation, resorption, and bone formation of mesenchymal stem cells (MSCs) [33, 34]. The decrease of serum IGF-1 protein has a significant relationship with osteoporosis, and strongly affects the incidence of osteoporotic fractures in postmenopausal women [11, 35, 36]. In addition, IGF-1 has also been confirmed in rat experiments to increase bone formation by stimulating the activity of osteoblasts and reduce bone resorption by limiting osteoclast differentiation [37]. In cartilage, *IGF-1* regulates chondrocyte differentiation and stimulates the synthesis of extracellular matrix components. In bone tissues, *IGF-1* enhances the function of differentiated osteoblasts and mediates parathyroid hormone-specific metabolic effects [38]. *IGF-1, PTH,* and *GH* have synergistic effects on osteogenesis. In postmenopausal women with hyperthyroidism, GH level is reduced, which decreases IGF-1 protein and causes osteogenesis [39]. Therefore, the role of *IGF-1* in relation to OP is diluted due to aging. Stratified by age at 70, we find that *IGF-1* SNPs in those with less than 70 show risky inclination but neutralization in those with more than 70. This result is shown in Supplementary Figure 2.

In the era of precision medicine, disease-associated genomic factors can be considered as biomarkers for disease. Machine learning models facilitate the identification of disease using biomarkers [40, 41]. As a result, the future prospect is to develop machine learning methods to identify the incidence or progression of OP.

Multiple tests in meta-analyses will result in p-value inflation and cause the study results to be significantly more easily, thereby increasing the probability of type I error occurring. TSA compares the confidence intervals of past meta-analyses and can better control type I and II errors [42]. The TSA results of this study showed that this study is a crucial sample, and a decisive conclusion is obtained from sufficient accumulated samples in rs35767.

There are some limitations in this study: 1. We only included English papers. It may lead to bias in this study. 2. This study population consists of Asians and cannot be generalized to other races.

## CONCLUSIONS

To conclude, our case-control study is a crucial sample in meta-analysis to reach conclusion of the association between IGF-1 rs35767 and OP in postmenopausal women.

## Supplementary Material

Supplementary Figures

Supplementary Table 1

Supplementary Table 2

Supplementary Tables 3 and 4
