# Supplementary material for: A case-control study coupling with meta-analysis elaborates decisive association between IGF-1 rs35767 and osteoporosis in Asian postmenopausal females
Source: Aging (Albany NY). 2023 Jan 3;15(1):134–47. doi: 10.18632/aging.204464 (PMC9876639; doi:10.18632/aging.204464)
Supplement: Supplementary Figures [file aging-15-204464-s001.pdf]

SUPPLEMENTARY FIGURES

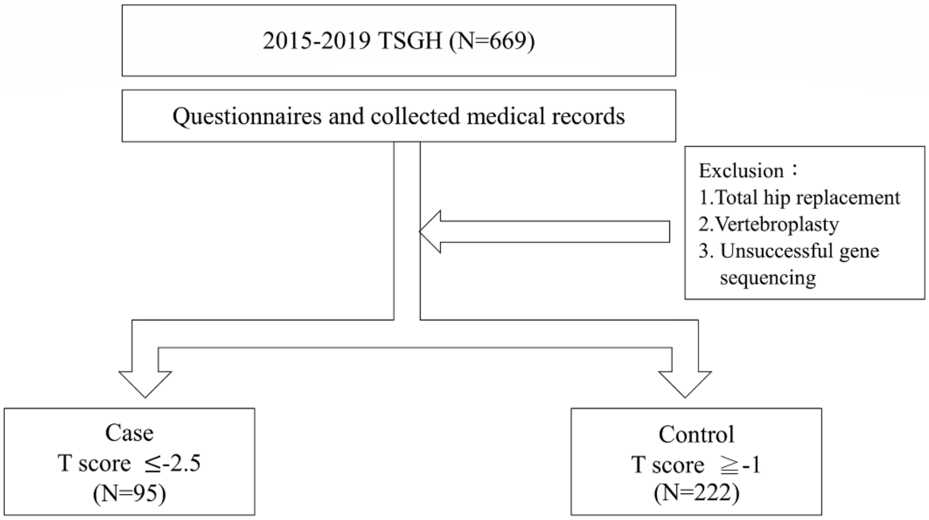

Supplementary Figure 1. Enrollment flowchart for case-control study

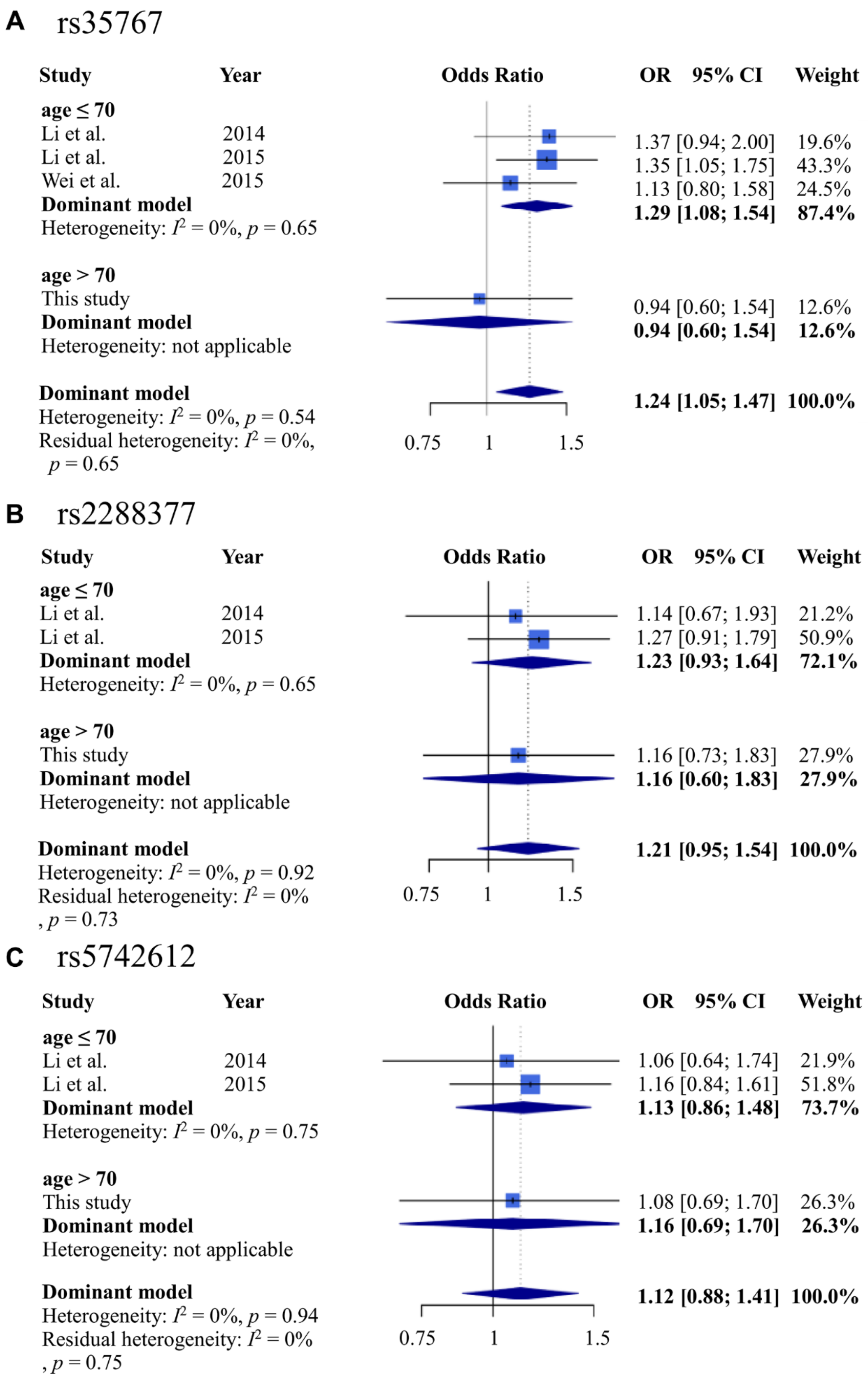

Supplementary Figure 2. Forest plot of the association between IGF-1 and OP stratified by age using dominant model. (**A**) The forest plot is based on rs35767 dominant model assumption (CT+TT vs. CC) stratified by age. (**B**) The forest plot is based on rs2288377 dominant model assumption (AT+TT vs AA) stratified by age. (**C**) The forest plot is based on rs5742612 dominant model assumption (TC+CC vs. TT) stratified by age.
