# Supplementary material for: A case-control study coupling with meta-analysis elaborates decisive association between IGF-1 rs35767 and osteoporosis in Asian postmenopausal females
Source: Aging (Albany NY). 2023 Jan 3;15(1):134–47. doi: 10.18632/aging.204464 (PMC9876639; doi:10.18632/aging.204464)
Supplement: Supplementary Table 2 [file aging-15-204464-s003.docx]

Supplementary Table 2. Related keywords and searching strategy

| **Relevant text of IGF-1** | **Relevant text of Osteoporosis** |
| --- | --- |
| 1. SNP | 1. Age-Related Osteoporosis |
| 1. SNPs | 1. Bone Loss, Age-Related |
| 1. genetic | 1. Osteoporosis, Age-Related |
| 1. polymorphism | 1. Osteoporosis, Involutional |
| 1. polymorphisms | 1. Osteoporosis, Post-Traumatic |
| 1. genetic polymorphisms | 1. Osteoporosis, Senile |
| 1. genetic polymorphism | 1. Senile Osteoporosis |
| 1. nucleotide polymorphism, single | **Combined (Final Strategy)** |
| 1. nucleotide polymorphisms, single | 1. (1 or 2 or 3 or 4 or 5 or 6 or 7 or 8 or 9 or 10 or 11) and (12 or 13 or 14 or 15 or 16 or 17 or 18 or 19 or 20 or 21) |
| 1. polymorphisms, single nucleotide |  |
| 1. single nucleotide polymorphisms |  |
| 1. single nucleotide polymorphism |  |
| 1. IGF-1 | 1. (22 or 23 or 24 or 25 or 26 or 27 or 28) |
| 1. IGF-I |  |
| 1. IGF-1-SmC | 1. 29 and 30 |
| 1. Insulin Like Growth Factor I |  |
| 1. Insulin-Like Somatomedin Peptide I |  |
| 1. Somatomedin C |  |
| 1. rs35767 |  |
| 1. rs2288377 |  |
| 1. rs5742612 |  |

MeSH Browser: http://www.nlm.nih.gov/mesh/MBrowser.html

PubMed: http://www.ncbi.nlm.nih.gov/pubmed

Cochrane Library: http://www.thecochranelibrary.com

Embase: https://www.embase.com

Web of science: <http://www.webofscience.com>

**Pubmed (105)**

1. Animal researches (10)[1-10]
2. Not related to this research (53)[11-63]
3. Not OP (26)[64-89]
4. Studies on other polymorphisms or gene (11)[90-100]
5. Not postmenopausal (2)[101, 102]
6. Included (3)[103-105]

**Cochrane (9)**

1. Duplication (1)[97]
2. Not related to this research (8)[106-113]

**Embase (58)**

1. Duplication (29) [4, 25, 27, 28, 32, 40, 41, 43, 50, 51, 57, 69, 76, 77, 81, 85, 88, 90, 92-96, 98, 100, 101, 103-105]
2. Animal research (1)[114]
3. Not related to this research (17)[115-131]
4. Not OP (3)[132-134]
5. Studies on other polymorphisms or gene (6) [135-140]
6. Poster (2)[141, 142]

**Web of Science (110)**

1. Duplication (57)[1-6, 9, 10, 15, 17, 18, 22, 25, 27, 31, 34, 35, 37-40, 42, 50, 54, 56-59, 65, 66, 69, 71, 73, 74, 76-78, 81, 84, 85, 87, 88, 90, 92, 95, 96, 98, 100-105, 118, 138, 141, 142]
2. Animal researches (12)[143-154]
3. Not related to this research (11)[155-165]
4. Not OP (24)[166-189]
5. Studies on other polymorphisms or gene (4)[190-193]
6. Not postmenopausal (2)[194, 195]

1. Cheng Y, Liu S, Zhang X, Wu Q, Li S, Fu H, Dong L, Yu H, Hao L. Expression Profiles of IGF-1R Gene and Polymorphisms of its Regulatory Regions in Different Pig Breeds. Protein J. 2016; 35:231-6.

2. Zhou H, Mitchell AD, McMurtry JP, Ashwell CM, Lamont SJ. Insulin-like growth factor-I gene polymorphism associations with growth, body composition, skeleton integrity, and metabolic traits in chickens. Poult Sci. 2005; 84:212-9.

3. Telldahl Y, Svensson E, Götherström A, Storå J. Typing late prehistoric cows and bulls--osteology and genetics of cattle at the Eketorp ringfort on the Öland island in Sweden. PLoS One. 2011; 6:e20748.

4. Bennett AK, Hester PY, Spurlock DE. Polymorphisms in vitamin D receptor, osteopontin, insulin-like growth factor 1 and insulin, and their associations with bone, egg and growth traits in a layer--broiler cross in chickens. Anim Genet. 2006; 37:283-6.

5. Hering DM, Olenski K, Kaminski S. Genome-wide association study for poor sperm motility in Holstein-Friesian bulls. Anim Reprod Sci. 2014; 146:89-97.

6. Gao X, Shin YH, Li M, Wang F, Tong Q, Zhang P. The fat mass and obesity associated gene FTO functions in the brain to regulate postnatal growth in mice. PLoS One. 2010; 5:e14005.

7. Kawai M, Delany AM, Green CB, Adamo ML, Rosen C J. Nocturnin suppresses igf1 expression in bone by targeting the 3' untranslated region of igf1 mRNA. Endocrinology. 2010; 151:4861-70.

8. Li JQ, Chen ZM, Liu DW, Liu XH, Sun BL, Ling F, Zhang H, Chen YS. Genetic effects of IGF-1 gene on the performance in Landrace x Lantang pig resource population. Yi Chuan Xue Bao. 2003; 30:835-9.

9. Wang X, Chrysovergis K, Bienstock RJ, Shim M, Eling TE. The H6D variant of NAG-1/GDF15 inhibits prostate xenograft growth in vivo. Prostate. 2012; 72:677-89.

10. McMichael BK, Jeong YH, Auerbach JA, Han CM, Sedlar R, Shettigar V, Bähler M, Agarwal S, Kim DG, Lee BS. The RhoGAP Myo9b Promotes Bone Growth by Mediating Osteoblastic Responsiveness to IGF-1. J Bone Miner Res. 2017; 32:2103-2115.

11. Park JH, Hwang IT, Yang S. Relationship between growth velocity and change of insulin-like growth factor-1, IGF binding protein-3 levels, and IGFBP-3 promoter polymorphism during GnRH agonist treatment. Ann Pediatr Endocrinol Metab. 2020.

12. Bang P. Pediatric Implications of Normal Insulin-GH-IGF-Axis Physiology, in Endotext, K.R. Feingold, et al., Editors. 2000, MDText.com, Inc.

Copyright © 2000-2020, MDText.com, Inc.: South Dartmouth (MA).

13. Tanaka T, Kato N, Aoki K, Watanabe M, Arai T, Hasegawa Y, Abe T. Long-term follow-up of growth hormone-producing pituitary carcinoma with multiple spinal metastases following multiple surgeries: case report. Neurol Med Chir (Tokyo). 2013; 53:707-11.

14. Westerbacka J, Bergholm R, Tiikkainen M, Yki-Järvinen H. Glargine and regular human insulin similarly acutely enhance endothelium-dependent vasodilatation in normal subjects. Arterioscler Thromb Vasc Biol. 2004; 24:320-4.

15. Meyer S, Haist M, Schaefer S, Ivan D, Ittner JR, Nawroth PP, Plöckinger U, Stalla GK, Tuschy U, Weber MM, Weise A, Pfützner A, Habbe N, et al. Association of COLIA1 Sp1 polymorphism with the effect of subcutaneously injected recombinant hGH in GH-deficient adults. Pharmacogenomics. 2008; 9:1017-26.

16. Teumer A, Qi Q, Nethander M, Aschard H, Bandinelli S, Beekman M, Berndt SI, Bidlingmaier M, Broer L, Cappola A, Ceda GP, Chanock S, Chen MH, et al. Genomewide meta-analysis identifies loci associated with IGF-I and IGFBP-3 levels with impact on age-related traits. Aging Cell. 2016; 15:811-24.

17. Liu XG, Tan LJ, Lei SF, Liu YJ, Shen H, Wang L, Yan H, Guo YF, Xiong DH, Chen XD, Pan F, Yang TL, Zhang YP, et al. Genome-wide association and replication studies identified TRHR as an important gene for lean body mass. Am J Hum Genet. 2009; 84:418-23.

18. Kaplan RC, Petersen AK, Chen MH, Teumer A, Glazer NL, Döring A, Lam C S, Friedrich N, Newman A, Müller M, Yang Q, Homuth G, Cappola A, et al. A genome-wide association study identifies novel loci associated with circulating IGF-I and IGFBP-3. Hum Mol Genet. 2011; 20:1241-51.

19. Pawlikowska L, Hu D, Huntsman S, Sung A, Chu C, Chen J, Joyner AH, Schork NJ, Hsueh WC, Reiner AP, Psaty BM, Atzmon G, Barzilai N, et al. Association of common genetic variation in the insulin/IGF1 signaling pathway with human longevity. Aging Cell. 2009; 8:460-72.

20. Lao JI, Montoriol C, Morer I, Beyer K. Genetic contribution to aging: deleterious and helpful genes define life expectancy. Ann N Y Acad Sci. 2005; 1057:50-63.

21. Cluett C, Melzer D. Human genetic variations: Beacons on the pathways to successful ageing. Mech Ageing Dev. 2009; 130:553-63.

22. Jiang M, Huhtaniemi I. Polymorphisms in androgen and estrogen receptor genes: effects on male aging. Exp Gerontol. 2004; 39:1603-11.

23. Chihara K, Takahashi Y, Kaji H, Goji K, Okimura Y, Abe H. Short stature caused by a natural growth hormone antagonist. Horm Res. 1998; 49 Suppl 1:41-5.

24. Juul A. The effects of oestrogens on linear bone growth. Hum Reprod Update. 2001; 7:303-13.

25. Dennison EM, Syddall HE, Jameson KA, Sayer AA, Gaunt TR, Rodriguez S, Day IN, Cooper C, Lips MA. A study of relationships between single nucleotide polymorphisms from the growth hormone-insulin-like growth factor axis and bone mass: the Hertfordshire cohort study. J Rheumatol. 2009; 36:1520-6.

26. Hamrick MW, Ferrari SL. Leptin and the sympathetic connection of fat to bone. Osteoporos Int. 2008; 19:905-12.

27. Kostek MC, Devaney JM, Gordish-Dressman H, Harris TB, Thompson PD, Clarkson PM, Angelopoulos TJ, Gordon PM, Moyna NM, Pescatello LS, Visich PS, Zoeller RF, Seip RL, et al. A polymorphism near IGF1 is associated with body composition and muscle function in women from the Health, Aging, and Body Composition Study. Eur J Appl Physiol. 2010; 110:315-24.

28. Bonjour JP, Chevalley T, Rizzoli R, Ferrari S. Gene-environment interactions in the skeletal response to nutrition and exercise during growth. Med Sport Sci. 2007; 51:64-80.

29. Smith SS, Kessler CB, Shenoy V, Rosen CJ, Delany AM. IGF-I 3' untranslated region: strain-specific polymorphisms and motifs regulating IGF-I in osteoblasts. Endocrinology. 2013; 154:253-62.

30. Millar DS, Horan M, Chuzhanova NA, Cooper DN. Characterisation of a functional intronic polymorphism in the human growth hormone (GH1) gene. Hum Genomics. 2010; 4:289-301.

31. Kato I, Eastham J, Li B, Smith M, Yu H. Genotype-phenotype analysis for the polymorphic CA repeat in the insulin-like growth factor-I (IGF-I) gene. Eur J Epidemiol. 2003; 18:203-9.

32. Takacs I, Koller DL, Peacock M, Christian JC, Hui SL, Conneally PM, Johnston CC Jr, Foroud T, Econs MJ. Sibling pair linkage and association studies between bone mineral density and the insulin-like growth factor I gene locus. J Clin Endocrinol Metab. 1999; 84:4467-71.

33. Shi Q, Rafii S, Wu MH, Wijelath ES, Yu C, Ishida A, Fujita Y, Kothari S, Mohle R, Sauvage LR, Moore MA, Storb RF, Hammond WP. Evidence for circulating bone marrow-derived endothelial cells. Blood. 1998; 92:362-7.

34. van Bunderen CC, Glad C, Johannsson G, Olsson DS. Personalized approach to growth hormone replacement in adults. Arch Endocrinol Metab. 2019; 63:592-600.

35. Feng C, Liu H, Yang Y, Huang B, Zhou Y. Growth and differentiation factor-5 contributes to the structural and functional maintenance of the intervertebral disc. Cell Physiol Biochem. 2015; 35:1-16.

36. Miyao M, Hosoi T, Ouchi Y. [Osteoporosis and genetic polymorphism]. Nihon Ronen Igakkai Zasshi. 1999; 36:245-50.

37. Gaudio A, Morabito N, Catalano A, Rapisarda R, Xourafa A, Lasco A. Pathogenesis of Thalassemia Major-associated Osteoporosis: A Review with Insights from Clinical Experience. J Clin Res Pediatr Endocrinol. 2019; 11:110-117.

38. Gao ST, Lv ZT, Zhou CK, Mao C, Sheng WB. Association between IGF-1 polymorphisms and risk of osteoporosis in Chinese population: a meta-analysis. BMC Musculoskelet Disord. 2018; 19:141.

39. Chen YC, Zhang L, Li EN, Ding LX, Zhang GA, Hou Y, Yuan W. Association of the insulin-like growth factor-1 single nucleotide polymorphisms rs35767, rs2288377, and rs5742612 with osteoporosis risk: A meta-analysis. Medicine (Baltimore). 2017; 96:e9231.

40. Niu T, Rosen CJ. The insulin-like growth factor-I gene and osteoporosis: a critical appraisal. Gene. 2005; 361:38-56.

41. Yadav A, Carey EJ. Osteoporosis in chronic liver disease. Nutr Clin Pract. 2013; 28:52-64.

42. Stagi S, Lapi E, Pantaleo M, Carella M, Petracca A, De Crescenzo A, Zelante L, Riccio A, de Martino M. A new case of de novo 6q24.2-q25.2 deletion on paternal chromosome 6 with growth hormone deficiency: a twelve-year follow-up and literature review. BMC Med Genet. 2015; 16:69.

43. Rizzoli R, Bonjour JP, Ferrari SL. Osteoporosis, genetics and hormones. J Mol Endocrinol. 2001; 26:79-94.

44. McKanna TA, Toriello HV. Gene doping: the hype and the harm. Pediatr Clin North Am. 2010; 57:719-27.

45. Guan M, Wang H, Fang H. Reply to the Letter to the Editor of Y. Zhao, et al. concerning "Association between IGF1 gene single nucleotide polymorphism (rs5742612) and adolescent idiopathic scoliosis: a meta-analysis" by Ming Guan, Huan Wang et al. Eur Spine J (2016). doi:10.1007/s00586-016-4742-7. Eur Spine J. 2017; 26:1314-1315.

46. Rosen CJ, Bilezikian JP. Perplexing polymorphisms: D(i)ps, Sn(i)ps, and trips. J Clin Endocrinol Metab. 1999; 84:4465-6.

47. Wit JM, Oostdijk W, Losekoot M, van Duyvenvoorde HA, Ruivenkamp CA, Kant S G. Mechanisms in Endocrinology: Novel genetic causes of short stature. Eur J Endocrinol. 2016; 174:R145-73.

48. Eisman JA. Genetics of osteoporosis. Endocr Rev. 1999; 20:788-804.

49. Backström MC, Mahonen A, Ala-Houhala M, Sievänen H, Mäenpää P, Koivisto AM, Olkku A, Mäki R, Mäki M. Genetic determinants of bone mineral content in premature infants. Arch Dis Child Fetal Neonatal Ed. 2001; 85:F214-6.

50. Esterle L, Sabatier JP, Guillon-Metz F, Walrant-Debray O, Guaydier-Souquières G, Jehan F, Garabédian M. Milk, rather than other foods, is associated with vertebral bone mass and circulating IGF-1 in female adolescents. Osteoporos Int. 2009; 20:567-75.

51. Baroncelli GI, Federico G, Bertelloni S, Ceccarelli C, Cupelli D, Saggese G. Vitamin-D receptor genotype does not predict bone mineral density, bone turnover, and growth in prepubertal children. Horm Res. 1999; 51:150-6.

52. Takahashi Y, Matsumoto M, Karasugi T, Watanabe K, Chiba K, Kawakami N, Tsuji T, Uno K, Suzuki T, Ito M, Sudo H, Minami S, Kotani T, et al. Lack of association between adolescent idiopathic scoliosis and previously reported single nucleotide polymorphisms in MATN1, MTNR1B, TPH1, and IGF1 in a Japanese population. J Orthop Res. 2011; 29:1055-8.

53. Mullis PE, Patel MS, Brickell PM, Hindmarsh PC, Brook CG. Growth characteristics and response to growth hormone therapy in patients with hypochondroplasia: genetic linkage of the insulin-like growth factor I gene at chromosome 12q23 to the disease in a subgroup of these patients. Clin Endocrinol (Oxf). 1991; 34:265-74.

54. Wang W, Luo XP, Cai LX, Cui ZR, Luo XY, Luo RK. Relationship between vitamin D receptor (VDR) polymorphisms and the efficacy of recombinant human growth hormone (rhGH) treatment in children with idiopathic short stature. Genet Mol Res. 2015; 14:10507-14.

55. Euser AM, Finken MJ, Kharagjitsingh AV, Alizadeh BZ, Roep BO, Meulenbelt I, Dekker FW, Wit JM. IGF1 promoter polymorphism and cranial growth in individuals born very preterm. Horm Res Paediatr. 2011; 76:27-34.

56. García RJ, Kant SG, Wit JM, Mericq V. Clinical and genetic characteristics and effects of long-term growth hormone therapy in a girl with Floating-Harbor syndrome. J Pediatr Endocrinol Metab. 2012; 25:207-12.

57. Diogenes ME, Bezerra FF, Cabello GM, Cabello PH, Mendonça LM, Oliveira Júnior AV, Donangelo CM. Vitamin D receptor gene FokI polymorphisms influence bone mass in adolescent football (soccer) players. Eur J Appl Physiol. 2010; 108:31-8.

58. d'Alésio A, Garabédian M, Sabatier JP, Guaydier-Souquières G, Marcelli C, Lemaçon A, Walrant-Debray O, Jehan F. Two single-nucleotide polymorphisms in the human vitamin D receptor promoter change protein-DNA complex formation and are associated with height and vitamin D status in adolescent girls. Hum Mol Genet. 2005; 14:3539-48.

59. Bezerra FF, Cabello GM, Mendonça LM, Donangelo CM. Bone mass and breast milk calcium concentration are associated with vitamin D receptor gene polymorphisms in adolescent mothers. J Nutr. 2008; 138:277-81.

60. Mysłek-Prucnal M, Bieniasz J, Noczyńska A. [Observation of children with total or partial somatotropic pituitary hypofunction treated with growth hormone (GH)]. Pediatr Endocrinol Diabetes Metab. 2010; 16:33-8.

61. Chang HP, Yang SF, Wang SL, Su PH. Associations among IGF-1, IGF2, IGF-1R, IGF-2R, IGFBP-3, insulin genetic polymorphisms and central precocious puberty in girls. BMC Endocr Disord. 2018; 18:66.

62. Lu L, Wang F, He L, Xue Y, Wang Y, Zhang H, Rong L, Wang M, Zhang Z, Fang Y, Miao H. Interaction Between IGF1 Polymorphisms and the Risk of Acute Lymphoblastic Leukemia in Chinese Children. Cell Physiol Biochem. 2015; 36:1346-58.

63. Silvano L, Miras M, Pérez A, Picotto G, Díaz de Barboza G, Muñoz L, Martin S, Sobrero G, Armelini P, Mericq V, Tolosa de Talamoni N. Comparative analysis of clinical, biochemical and genetic aspects associated with bone mineral density in small for gestational age children. J Pediatr Endocrinol Metab. 2011; 24:511-7.

64. Michl P, Spoettl G, Engelhardt D, Weber MM. Alterations of the insulin-like growth factor system in patients with polycythemia vera. Mol Cell Endocrinol. 2001; 181:189-97.

65. Lim SY, Zalilah MS, Chin YS, Ramachandran V, Chan YM. Dietary Acid Load, IGF-1 Single Nucleotide Polymorphism and Bone Resorption among Postmenopausal Chinese Women. Nutrients. 2018; 10.

66. Mao J, Zhuang G, Chen Z. Genetic Polymorphisms of Insulin-Like Growth Factor 1 Are Associated with Osteosarcoma Risk and Prognosis. Med Sci Monit. 2017; 23:5892-5898.

67. Kushlinskii NE, Tivofeev YS, Generozov EV, Naumov VA, Soloviev YN, Boulytcheva IV, Aliev MD. Associations of single nucleotide polymorphisms with malignant and borderline bone tumors. Klin Lab Diagn. 2013; 10:58-60, 22-4.

68. Reimann E, Kõks S, Ho XD, Maasalu K, Märtson A. Whole exome sequencing of a single osteosarcoma case--integrative analysis with whole transcriptome RNA-seq data. Hum Genomics. 2014; 8:20.

69. Chiloiro S, Mormando M, Bianchi A, Giampietro A, Milardi D, Bima C, Grande G, Formenti AM, Mazziotti G, Pontecorvi A, Giustina A, De Marinis L. Prevalence of morphometric vertebral fractures in "difficult" patients with acromegaly with different biochemical outcomes after multimodal treatment. Endocrine. 2018; 59:449-453.

70. Wassenaar MJ, Biermasz NR, Pereira AM, van der Klaauw AA, Smit JW, Roelfsema F, van der Straaten T, Cazemier M, Hommes DW, Kroon HM, Kloppenburg M, Guchelaar HJ, Romijn JA. The exon-3 deleted growth hormone receptor polymorphism predisposes to long-term complications of acromegaly. J Clin Endocrinol Metab. 2009; 94:4671-8.

71. Tsuchiya N, Narita S, Inoue T, Saito M, Numakura K, Huang M, Hatakeyama S, Satoh S, Saito S, Ohyama C, Arai Y, Ogawa O, Habuchi T. Insulin-like growth factor-1 genotypes and haplotypes influence the survival of prostate cancer patients with bone metastasis at initial diagnosis. BMC Cancer. 2013; 13:150.

72. Tsuchiya N, Wang L, Suzuki H, Segawa T, Fukuda H, Narita S, Shimbo M, Kamoto T, Mitsumori K, Ichikawa T, Ogawa O, Nakamura A, Habuchi T. Impact of IGF-I and CYP19 gene polymorphisms on the survival of patients with metastatic prostate cancer. J Clin Oncol. 2006; 24:1982-9.

73. Song Y, Du Z, Yang Q, Ren M, Sui Y, Wang Q, Wang A, Zhao H, Wang J, Zhang G. Associations of IGFBP3 Gene Polymorphism and Gene Expression with the Risk of Osteonecrosis of the Femoral Head in a Han Population in Northern China. DNA Cell Biol. 2016; 35:836-844.

74. Urano T, Narusawa K, Shiraki M, Usui T, Sasaki N, Hosoi T, Ouchi Y, Nakamura T, Inoue S. Association of a single nucleotide polymorphism in the insulin-like growth factor-1 receptor gene with spinal disc degeneration in postmenopausal Japanese women. Spine (Phila Pa 1976). 2008; 33:1256-61.

75. Lakatos PL, Bajnok E, Tornai I, Folhoffer A, Horváth A, Lakatos P, Szalay F. [Decreased bone mineral density and gene polymorphism in primary biliary cirrhosis]. Orv Hetil. 2004; 145:331-6.

76. Lakatos PL, Bajnok E, Tornai I, Folhoffer A, Horvath A, Lakatos P, Habior A, Szalay F. Insulin-like growth factor I gene microsatellite repeat, collagen type Ialpha1 gene Sp1 polymorphism, and bone disease in primary biliary cirrhosis. Eur J Gastroenterol Hepatol. 2004; 16:753-9.

77. Rivadeneira F, Houwing-Duistermaat JJ, Beck TJ, Janssen JA, Hofman A, Pols HA, Van Duijn CM, Uitterlinden AG. The influence of an insulin-like growth factor I gene promoter polymorphism on hip bone geometry and the risk of nonvertebral fracture in the elderly: the Rotterdam Study. J Bone Miner Res. 2004; 19:1280-90.

78. Szczęsny G, Olszewski WL, Zagozda M, Rutkowska J, Czapnik Z, Swoboda-Kopeć E, Górecki A. Genetic factors responsible for long bone fractures non-union. Arch Orthop Trauma Surg. 2011; 131:275-81.

79. Martín S, Muñoz L, Pérez A, Sobrero G, Picotto G, Ochetti M, Carpentieri A, Silvano L, de Barboza GD, Signorino M, Rupérez C, Bertolotto P, Ulla MR, et al. Clinical and molecular studies related to bone metabolism in patients with congenital adrenal hyperplasia. J Pediatr Endocrinol Metab. 2014; 27:1161-6.

80. Meulenbelt I, Bijkerk C, Miedema HS, Breedveld FC, Hofman A, Valkenburg HA, Pols HA, Slagboom PE, van Duijn CM. A genetic association study of the IGF-1 gene and radiological osteoarthritis in a population-based cohort study (the Rotterdam Study). Ann Rheum Dis. 1998; 57:371-4.

81. Zhai G, Rivadeneira F, Houwing-Duistermaat JJ, Meulenbelt I, Bijkerk C, Hofman A, van Meurs JB, Uitterlinden AG, Pols HA, Slagboom PE, van Duijn CM. Insulin-like growth factor I gene promoter polymorphism, collagen type II alpha1 (COL2A1) gene, and the prevalence of radiographic osteoarthritis: the Rotterdam Study. Ann Rheum Dis. 2004; 63:544-8.

82. Dresner Pollack R, Rachmilewitz E, Blumenfeld A, Idelson M, Goldfarb AW. Bone mineral metabolism in adults with beta-thalassaemia major and intermedia. Br J Haematol. 2000; 111:902-7.

83. Griffiths AM. Growth retardation in early-onset inflammatory bowel disease: should we monitor and treat these patients differently? Dig Dis. 2009; 27:404-11.

84. Whyte MP, Totty WG, Novack DV, Zhang X, Wenkert D, Mumm S. Camurati-Engelmann disease: unique variant featuring a novel mutation in TGFβ1 encoding transforming growth factor beta 1 and a missense change in TNFSF11 encoding RANK ligand. J Bone Miner Res. 2011; 26:920-33.

85. Tirabassi G, delli Muti N, Gioia A, Biagioli A, Lenzi A, Balercia G. Effects of testosterone replacement therapy on bone metabolism in male post-surgical hypogonadotropic hypogonadism: focus on the role of androgen receptor CAG polymorphism. J Endocrinol Invest. 2014; 37:393-400.

86. Hess O, Hujeirat Y, Wajnrajch MP, Allon-Shalev S, Zadik Z, Lavi I, Tenenbaum-Rakover Y. Variable phenotypes in familial isolated growth hormone deficiency caused by a G6664A mutation in the GH-1 gene. J Clin Endocrinol Metab. 2007; 92:4387-93.

87. Su PH, Chen JY, Yu JS, Chen SJ, Yang SF. Leptin expression and leptin receptor gene polymorphisms in growth hormone deficiency patients. Hum Genet. 2011; 129:455-62.

88. Furushima K, Shimo-Onoda K, Maeda S, Nobukuni T, Ikari K, Koga H, Komiya S, Nakajima T, Harata S, Inoue I. Large-scale screening for candidate genes of ossification of the posterior longitudinal ligament of the spine. J Bone Miner Res. 2002; 17:128-37.

89. Yeung HY, Tang NL, Lee KM, Ng BK, Hung VW, Kwok R, Guo X, Qin L, Cheng JC. Genetic association study of insulin-like growth factor-I (IGF-I) gene with curve severity and osteopenia in adolescent idiopathic scoliosis. Stud Health Technol Inform. 2006; 123:18-24.

90. Koromila T, Georgoulias P, Dailiana Z, Ntzani EE, Samara S, Chassanidis C, Aleporou-Marinou V, Kollia P. CER1 gene variations associated with bone mineral density, bone markers, and early menopause in postmenopausal women. Hum Genomics. 2013; 7:21.

91. Duncan EL, Brown MA, Sinsheimer J, Bell J, Carr AJ, Wordsworth BP, Wass J A. Suggestive linkage of the parathyroid receptor type 1 to osteoporosis. J Bone Miner Res. 1999; 14:1993-9.

92. Lee DO, Jee BC, Ku SY, Suh CS, Kim SH, Choi YM, Moon SY, Kim JG. Relationships between the insulin-like growth factor I (IGF-I) receptor gene G3174A polymorphism, serum IGF-I levels, and bone mineral density in postmenopausal Korean women. J Bone Miner Metab. 2008; 26:42-6.

93. Kim JG, Roh KR, Lee JY. The relationship among serum insulin-like growth factor-I, insulin-like growth factor-I gene polymorphism, and bone mineral density in postmenopausal women in Korea. Am J Obstet Gynecol. 2002; 186:345-50.

94. Miyao M, Hosoi T, Inoue S, Hoshino S, Shiraki M, Orimo H, Ouchi Y. Polymorphism of insulin-like growth factor I gene and bone mineral density. Calcif Tissue Int. 1998; 63:306-11.

95. Rivadeneira F, Houwing-Duistermaat JJ, Vaessen N, Vergeer-Drop JM, Hofman A, Pols HA, Van Duijn CM, Uitterlinden AG. Association between an insulin-like growth factor I gene promoter polymorphism and bone mineral density in the elderly: the Rotterdam Study. J Clin Endocrinol Metab. 2003; 88:3878-84.

96. Jiang DK, Shen H, Li MX, Jiang C, Yang N, Zhu J, Wu Y, Qin YJ, Zhou Q, Deng HW. No major effect of the insulin-like growth factor I gene on bone mineral density in premenopausal Chinese women. Bone. 2005; 36:694-9.

97. Rosen CJ, Kurland ES, Vereault D, Adler RA, Rackoff PJ, Craig WY, Witte S, Rogers J, Bilezikian JP. Association between serum insulin growth factor-I (IGF-I) and a simple sequence repeat in IGF-I gene: implications for genetic studies of bone mineral density. J Clin Endocrinol Metab. 1998; 83:2286-90.

98. Rivadeneira F, van Meurs JB, Kant J, Zillikens MC, Stolk L, Beck TJ, Arp P, Schuit SC, Hofman A, Houwing-Duistermaat JJ, van Duijn CM, van Leeuwen JP, Pols HA, et al. Estrogen receptor beta (ESR2) polymorphisms in interaction with estrogen receptor alpha (ESR1) and insulin-like growth factor I (IGF1) variants influence the risk of fracture in postmenopausal women. J Bone Miner Res. 2006; 21:1443-56.

99. Mezquita-Raya P, Muñoz-Torres M, Alonso G, de Luna JD, Quesada JM, Dorado G, Luque-Recio F, Ruiz-Requena ME, Lopez-Rodriguez F, Escobar-Jiménez F. Susceptibility for postmenopausal osteoporosis: interaction between genetic, hormonal and lifestyle factors. Calcif Tissue Int. 2004; 75:373-9.

100. Sheu YT, Zmuda JM, Cauley JA, Moffett SP, Rosen CJ, Ishwad C, Ferrell RE. Nuclear receptor coactivator-3 alleles are associated with serum bioavailable testosterone, insulin-like growth factor-1, and vertebral bone mass in men. J Clin Endocrinol Metab. 2006; 91:307-12.

101. Zhang W, Zhang LC, Chen H, Tang PF, Zhang LH. Association between polymorphisms in insulin-like growth factor-1 and risk of osteoporosis. Genet Mol Res. 2015; 14:7655-60.

102. Fan Y, Zhang S, Liang F, Zhou Y. Association of insulin-like growth factor I gene polymorphisms with the risk of osteoporosis in a Chinese population. Int J Clin Exp Pathol. 2017; 10:8443-8451.

103. Yun-Kai L, Hui W, Xin-Wei Z, Liang G, Jin-Liang Z. The polymorphism of Insulin-like growth factor-I (IGF-I) is related to osteoporosis and bone mineral density in postmenopausal population. Pak J Med Sci. 2014; 30:131-5.

104. Li F, Xing WH, Yang XJ, Jiang HY, Xia H. Influence of polymorphisms in insulin-like growth factor-1 on the risk of osteoporosis in a Chinese postmenopausal female population. Int J Clin Exp Pathol. 2015; 8:5727-32.

105. Wei YK, Ma HL, Guo YZ, Yang BH, Pang WX. Association of the IGF-1 rs35767 and rs972936 polymorphisms with the risk of osteoporosis in a Chinese postmenopausal female population. Genet Mol Res. 2015; 14:14325-30.

106. Huang YT, Lin X, Chirieac LR, McGovern R, Wain JC, Heist RS, Skaug V, Zienolddiny S, Haugen A, Su L, et al. Cigarette smoking increases copy number alterations in non-small cell lung cancer. Cancer research. 2010; 70.

107. Euctr AT. Vitamin D and testosterone in men. <http://www.who.int/trialsearch/Trial2.aspx?TrialID=EUCTR2011-003575-11-AT>. 2013.

108. Bartell EB, Fujimoto M, Khoury PR, Vedantam S, Astley CM, Hirschhorn JN, Dauber AD. Protein QTL analysis of IGF-I and its binding proteins provides insights into growth biology. Human molecular genetics. 2020; 29:2625-36.

109. Nct. Bone Density in Children With IBD Treated With Amorphous Calcium or Commercial Crystalline Calcium. <https://clinicaltrials.gov/show/NCT02470663>. 2015.

110. Nct. Influence of External Factors on Skeletal Growth in Youth. <https://clinicaltrials.gov/show/NCT00633828>. 2008.

111. Van De Ven S, Liefers GJ, Putter H, Van Warmerdam LJ, Kessels LW, Dercksen W, Pepels MJ, Maartense E, Van Laarhoven HWM, Vriens B, et al. NEO-ZOTAC: toxicity data of a phase III randomized trial with NEOadjuvant chemotherapy (TAC) with or without ZOledronic acid (ZA) for patients with HER2-negative large resectable or locally advanced breast cancer (BC). Cancer research. 2012; 72.

112. Crew KD, Lew DL, Hershman DL, Refice S, Anderson GL, Hortobagyi GN, Goodman GE, Brown PH. Phase IIB randomized double-blind placebo-controlled biomarker modulation study of high dose vitamin D in premenopausal women at high-risk for breast cancer: SWOG S0812. Cancer research. 2013; 73.

113. Bright GM, Miller BS, Galvan M, Ng D, Humphriss E, Charlton RW. IGF family biomarkers in the diagnosis of pediatric growth hormone deficiency (PGHD) in somavaratan clinical trials. Endocrine reviews. Conference: 99th annual meeting of the endocrine society, ENDO 2017. United states. 2017; 38.

114. Mohan S, Masinde G, Li X, Baylink DJ. Mapping quantitative trait loci that influence serum insulin-like growth factor binding protein-5 levels in F2 mice (MRL/MpJ X SJL/J). Endocrinology. 2003; 144:3491-3496.

115. Giroux S, Elfassihi L, Clément V, Bussières J, Bureau A, Cole DEC, Rousseau F. High-density polymorphisms analysis of 23 candidate genes for association with bone mineral density. Bone. 2010; 47:975-981.

116. Sims NA. Building bone with a SOST-PTH partnership. Journal of Bone and Mineral Research. 2010; 25:175-177.

117. Wright NM, Renault J, Willi S, Veldhuis JD, Pandey JP, Gordon L, Key LL, Bell NH. Greater secretion of growth hormone in black than in white men: Possible factor in greater bone mineral density - A Clinical Research Center Study. J Clin Endocrinol Metab. 1995; 80:2291-2297.

118. Díaz De Barboza G, Pérez A, Martín S, Miras M, Picotto G, Muñoz L, Carpentieri A, Sobrero G, Ochetti M, Silvano L, Signorino M, Rupérez C, Bertolotto P, et al. Association between bone mineral density and VDR and IGF-1 gene polymorphisms in patients with congenital adrenal hyperplasia treated with glucocorticoids. Bone. 2013; 53:599.

119. Picotto G, Martín S, Muñóz L, Perez A, Díaz De Barboza G, Sobrero G, Ochetti M, Carpentieri A, Silvano L, Signorino M, Ruperez C, Bertolotto P, Pellizas C, et al. Association between bone mineral density and VDR and IGF-1 genes polymorphisms in patients with congenital adrenal hyperplasia (CAH-21OH) treated with glucocorticoids. Hormone Research in Paediatrics. 2012; 78:36.

120. Audí L, García-Ramírez M, Carrascosa A. Genetic determinants of bone mass. Hormone Research. 1999; 51:105-123.

121. Rubin K. Pubertal development and bone. C Curr Opin Endocrinol Diabetes. 2000; 7:65-70.

122. Harvey N, Arden N. The epidemiology of osteoporotic fractures. CPD Bulletin Clinical Biochemistry. 2003; 5:35-40.

123. Takács I, Lakatos P. Genetic background of osteoporosis. Orvosi hetilap. 2003; 144:1467-1472.

124. Xiu CY, Guo JH, Cai H. Candidate gene polymorphisms of primary osteoporosis. Chinese Journal of Clinical Rehabilitation. 2005; 9:138-140.

125. Ferrari S. Human genetics of osteoporosis. Best Pract Res Clin Endocrinol Metab. 2008; 22:723-735.

126. Cooper C, Harvey N, Cole Z, Hanson M, Dennison E. Developmental origins of osteoporosis: The role of maternal nutrition. Adv Exp Med Biol. 2009; 646:31-39.

127. Miškić B, Meandžija I, Ivanko M, Miškić D, Ćosić V, Mirošević G. Genetic polymorphism associated with ultrasound indicators of the calcaneal bone mineral density in children. International Journal of Rheumatic Diseases. 2012; 15:40.

128. Pédiatriques E. Somatropic axis adaptation during pubertal development F Brioude Explorations Fonctionnelles. Fundamental and Clinical Pharmacology. 2012; 26:8.

129. Eriksson AL, Suuriniemi M, Mahonen A, Cheng S, Ohlsson C. The COMT val158met polymorphism is associated with early pubertal development, height and cortical bone mass in girls. Pediatric Research. 2005; 58:71-77.

130. Khosla S, Bellido TM, Drezner MK, Gordon CM, Harris TB, Kiel DP, Kream BE, Leboff MS, Lian JB, Peterson CA, Rosen CJ, Williams JP, Winer KK, et al. Forum on aging and skeletal health: Summary of the proceedings of an ASBMR workshop. Journal of Bone and Mineral Research. 2011; 26:2565-2578.

131. Pollak RD, Rachmilewitz E, Blumenfeld A, Idelson M, Goldfarb AW. Bone mineral metabolism in adults with β-thalassaemia major and intermedia. British Journal of Haematology. 2000; 111:902-907.

132. Mormando M, Nasto LA, Bianchi A, Mazziotti G, Giampietro A, Pola E, Pontecorvi A, Giustina A, De Marinis L. GH receptor isoforms and skeletal fragility in acromegaly. European Journal of Endocrinology. 2014; 171:237-245.

133. Qiu XS, Tang NLS, Yeung HY, Qiu Y, Cheng JCY. Genetic association study of growth hormone receptor and idiopathic scoliosis. Clinical Orthopaedics and Related Research. 2007:53-58.

134. Parés A, Guañabens N, Rodés J. Gene polymorphisms as predictors of decreased bone mineral density and osteoporosis in primary biliary cirrhosis. Eur J Gastroenterol Hepatol. 2005; 17:311-315.

135. Thijssen J. Gene polymorphisms involved in the regulation of bone quality. Gynecological Endocrinology. 2006; 22:131-139.

136. Canhao H, Fonseca JE, Caetano-Lopes J, Saldanha C, Queiroz MV. Assessment of laboratory measurements and -308 TNFα gene promoter polymorphisms in normal bone mineral density. Clinical Rheumatology. 2008; 27:301-307.

137. Riancho JA. Role of the klotho gene in bone and mineral metabolism. Clinical Reviews in Bone and Mineral Metabolism. 2008; 6:31-36.

138. Liu SL, Lei SF, Yang F, Li X, Liu R, Nie S, Liu X G, Yang T L, Guo Y, Deng F Y, Tian Q, Li J, Liu YZ, et al. Copy number variation in CNP267 region may be associated with hip bone size. PLoS ONE. 2011; 6.

139. Wang Y, Guo TK, Liu J, Yang RF, Shao FF, Tian LM. Association of insulin-like growth factor 1 receptor gene rs2229765 polymorphism with osteoporosis in postmenopausal women. Chinese Journal of Tissue Engineering Research. 2017; 21:1813-1818.

140. Li GHY, Deng HW, Kung AWC, Huang QY. Identification of genes for bone mineral density variation by computational disease gene identification strategy. Journal of Bone and Mineral Metabolism. 2011; 29:709-716.

141. Xu W, Wang G. No association of rs35767 polymorphism of IGF-1 with bone mineral density in chinese postmenopausal women. Osteoporosis International. 2012; 23:S801.

142. Mohamed H, Ismail A, Braima K. Effect of IGF-1 gene polymorphism on bone mineral density in osteoporotic postmenopausal sudanese women. Osteoporosis International. 2019; 30:S647.

143. Wang CL, Wang SY, Liu SC, Cheng YN, Geng HW, Yang R, Feng TQ, Lu GH, Sun XT, Song J, Hao LN. Synonymous Mutations of PorcineIgf1rExtracellular Domain Affect Differentiation and Mineralization in MC3T3-E1 Cells. Frontiers in Cell and Developmental Biology. 2020; 8.

144. Ma YL, Wen YF, Cao XK, Cheng J, Huang YZ, Ma Y, Hu LY, Lei CZ, Qi XL, Cao H, Chen H. Copy number variation (CNV) in the IGF1R gene across four cattle breeds and its association with economic traits. Arch Anim Breed. 2019; 62:171-179.

145. Huang JM, Zhao Q, Wang KJ, Chen C, Xie SL, Liu DD. Effects of zoledronic acid on osteoblast proliferation and IGF-1 expression in rats in vitro. Farmacia. 2019; 67:154-159.

146. Naicy T, Venkatachalapathy T, Aravindakshan T, Raghavan K C, Mini M, Shyama K. Association of a novel single nucleotide polymorphism at the exon-2 of Insulin-Like Growth Factor 1 (IGF1) gene with phenotypic variants in goats. Veterinarski Arhiv. 2017; 87:457-472.

147. Wang YC, Jiang RR, Kang XT, Li ZJ, Han RL, Geng J, Fu JX, Wang JF, Wu JP. Identification of single nucleotide polymorphisms in the ASB15 gene and their associations with chicken growth and carcass traits. Genetics and Molecular Research. 2015; 14:11377-11388.

148. Chen A, Hao LL, Fang XB, Lu K, Liu SC, Zhang YL. Polymorphism analysis of IGFBP-5 gene exon 1 in Tibet Mini-pig and Junmu No. 1 White pig. Genetics and Molecular Research. 2014; 13:1643-1649.

149. Sharma A, Dutt G, Sivalingam J, Singh MK, Pathodiya OP, Khadda BS, Dixit SP. Novel SNPs in IGF1, GHR and IGFBP-3 genes reveal significant association with growth traits in Indian goat breeds. Small Ruminant Research. 2013; 115:7-14.

150. Boschiero C, Jorge EC, Ninov K, Nones K, do Rosario MF, Coutinho LL, Ledur MC, Burt DW, Moura A. Association of IGF1 and KDM5A polymorphisms with performance, fatness and carcass traits in chickens. Journal of Applied Genetics. 2013; 54:103-112.

151. Adamis D, Treloar A, Gregson N, Macdonald AJD, Martin FC. Delirium and the functional recovery of older medical inpatients after acute illness: The significance of biological factors. Archives of Gerontology and Geriatrics. 2011; 52:276-280.

152. Kurosu H, Yamamoto M, Clark JD, Pastor JV, Nandi A, Gurnani P, McGuinness OP, Chikuda H, Yamaguchi M, Kawaguchi H, Shimomura I, Takayama Y, Herz J, et al. Suppression of aging in mice by the hormone Klotho. Science. 2005; 309:1829-1833.

153. Ge W, Davis ME, Hines HC, Irvin KM, Simmen RCM. Association of a genetic marker with blood serum insulin-like growth factor-I concentration and growth traits in Angus cattle. Journal of Animal Science. 2001; 79:1757-1762.

154. Tao L, He XY, Wang FY, Zhong YJ, Pan LX, Wang XY, Gan SQ, Di R, Chu M X. Luzhong mutton sheep: inbreeding and selection signatures. Journal of Animal Science and Technology. 2020; 62:777-789.

155. Puthucheary Z, Skipworth JRA, Rawal J, Loosemore M, Van Someren K, Montgomery HE. Genetic Influences in Sport and Physical Performance. Sports Medicine. 2011; 41:845-859.

156. Vanderschueren D, Pye SR, Venken K, Borghs H, Gaytant J, Huhtaniemi IT, Adams JE, Ward KA, Bartfai G, Casanueva FF, Finn JD, Forti G, Giwercman A, et al. Gonadal sex steroid status and bone health in middle-aged and elderly European men. Osteoporosis International. 2010; 21:1331-1339.

157. Sweeney C, Murtaugh MA, Baumgartner KB, Byers T, Giuliano AR, Herrick JS, Wolff R, Caan BJ, Slattery ML. Insulin-like growth factor pathway polymorphisms associated with body size in hispanic and non-hispanic white women. Cancer Epidemiol Biomarkers Prev. 2005; 14:1802-1809.

158. Kostek MC, Delmonico MJ, Reichel JB, Roth SM, Douglass L, Ferrell RE, Hurley BF. Muscle strength response to strength training is influenced by insulin-like growth factor 1 genotype in older adults. Journal of Applied Physiology. 2005; 98:2147-2154.

159. Galle SA, Geraedts IK, Deijen JB, Milders MV, Drent ML. The Interrelationship between Insulin-Like Growth Factor 1, Apolipoprotein E epsilon 4, Lifestyle Factors, and the Aging Body and Brain. J Prev Alzheimers Dis. 2020; 7:265-273.

160. Yang TL, Xiong DH, Guo Y, Recker RR, Deng HW. Comprehensive association analyses of IGF1, ESR2, and CYP17 genes with adult height in Caucasians. European Journal of Human Genetics. 2008; 16:1380-1387.

161. Jensen RBB, Chellakootya M, Vielwerth S, Vaag A, Larsen T, Greisen G, Skakkebaek NE, Scheike T, Juul A. Intrauterine growth retardation and consequences for endocrine and cardiovascular diseases in adult life: Does insulin-like growth factor-I play a role? Hormone Research. 2003; 60:136-148.

162. Laurent M, Antonio L, Sinnesael M, Dubois V, Gielen E, Classens F, Vanderschueren D. Androgens and estrogens in skeletal sexual dimorphism. Asian Journal of Andrology. 2014; 16:213-222.

163. Fletcher O, Gibson L, Johnson N, Altmann DR, Holly JMP, Ashworth A, Peto J, Silva ID. Polymorphisms and circulating levels in the insulin-like growth factor system and risk of breast cancer: A systematic review. Cancer Epidemiol Biomarkers Prev. 2005; 14:2-19.

164. Paroni G, Seripa D, Panza F, Addante F, Copetti M, D'Onofrio G, Pellegrini F, Fontana L, Pilotto A. Klotho locus, metabolic traits, and serum hemoglobin in hospitalized older patients: a genetic association analysis. Age. 2012; 34:949-968.

165. Balci B, Yildiz B O, Ofir R, Dincer P, Bayraktar M. Linkage Analysis in a Large Primary Osteoporosis Family. Turkish Journal of Biochemistry-Turk Biyokimya Dergisi. 2008; 33:215-222.

166. Dayan CM, Panicker V. Novel insights into thyroid hormones from the study of common genetic variation. Nat Rev Endocrinol. 2009; 5:211-218.

167. Chia VM, Sakoda LC, Graubard BI, Rubertone MV, Chanock SJ, Erickson RL, McGlynn KA. Risk of testicular germ cell tumors and polymorphisms in the insulin-like growth factor genes. Cancer Epidemiol Biomarkers Prev. 2008; 17:721-726.

168. Perry JK, Wu ZS, Mertani HC, Zhu T, Lobie PE. Tumour-Derived Human Growth Hormone As a Therapeutic Target in Oncology. Trends in Endocrinology and Metabolism. 2017; 28:587-596.

169. Savage SA, Woodson K, Walk E, Modi W, Liao J, Douglass C, Hoover RN, Chanock SJ, and National Osteosarcoma Etiology Study Group. Analysis of genes critical for growth regulation identifies insulin-like growth factor 2 receptor variations with possible functional significance as risk factors for osteosarcoma. Cancer Epidemiol Biomarkers Prev. 2007; 16:1667-1674.

170. Xie H, Mao JS, Hu WF. Insulin-Like Growth Factor 1 (IGF1) Pathway Member Polymorphisms Are Associated with Risk and Prognosis of Chondrosarcoma. Medical Science Monitor. 2020; 26.

171. Pechlivanis S, Wagner K, Chang-Claude J, Hoffmeister M, Brenner H, Forsti A. Polymorphisms in the insulin like growth factor 1 and IGF binding protein 3 genes and risk of colorectal cancer. Cancer Detection and Prevention. 2007; 31:408-416.

172. Keku TO, Vidal A, Oliver S, Hoyo C, Hall IJ, Omofoye O, McDoom M, Worley K, Galanko J, Sandler RS, Millikan R. Genetic variants in IGF-I, IGF-II, IGFBP-3, and adiponectin genes and colon cancer risk in African Americans and Whites. Cancer Causes & Control. 2012; 23:1127-1138.

173. Schildkraut JM, Demark-Wahnefried W, Wenham RM, Grubber J, Jeffreys AS, Grambow SC, Marks JR, Moorman PG, Hoyo C, Ali S, Walther PJ. IGF1 (CA)(19) repeat and IGFBP3-202 A/C genotypes and the risk of prostate cancer in black and white men. Cancer Epidemiol Biomarkers Prev. 2005; 14:403-408.

174. Tsuchiya N, Wang LH, Horikawa Y, Inoue T, Kakinuma H, Matsuura S, Sato K, Ogawa O, Kato T, Habuchi T. CA repeat polymorphism in the insulin-like growth factor-I gene is associated with increased risk of prostate cancer and benign prostatic hyperplasia. International Journal of Oncology. 2005; 26:225-231.

175. Peehl DM, Krishnan AV, Feldman D. Pathways mediating the growth-inhibitory actions of vitamin D in prostate cancer. J Nutr. 2003; 133:2461S-2469S.

176. Jernstrom H, Chu M, Vesprini D, Tao YZ, Majeed N, Deal C, Pollak M, Narod SA. Genetic factors related to racial variation in plasma levels of insulin-like growth factor-1: Implications for premenopausal breast cancer risk. Molecular Genetics and Metabolism. 2001; 72:144-154.

177. Gee J, Bailey H, Kim K, Kolesar J, Havighurst T, Tutsch K D, See W, Cohen M B, Street N, LeVan L, Jarrard D, Wilding G. Phase II open label, multi-center clinical trial of modulation of intermediate endpoint biomarkers by 1-hydroxyvitamin D2 in patients with clinically localized prostate cancer and high grade pin. Prostate. 2013; 73:970-978.

178. Dumitrescu RG, Cotarla I. Understanding breast cancer risk - where do we stand in 2005? Journal of Cellular and Molecular Medicine. 2005; 9:208-221.

179. Yu H, Li BDL, Smith M, Shi R, Berkel HJ, Kato I. Polymorphic CA repeats in the IGF-I gene and breast cancer. Breast Cancer Research and Treatment. 2001; 70:117-122.

180. Marzec M, Hawkes CP, Eletto D, Boyle S, Rosenfeld R, Hwa V, Wit JM, van Duyvenvoorde HA, Oostdijk W, Losekoot M, Pedersen O, Yeap BB, Flicker L, et al. A Human Variant of Glucose-Regulated Protein 94 That Inefficiently Supports IGF Production. Endocrinology. 2016; 157:1914-1928.

181. Morimoto LM, Newcomb PA, White E, Bigler J, Potter JD. Variation in plasma insulin-like growth factor-1 and insulin-like growth factor binding protein-3: Genetic factors. Cancer Epidemiol Biomarkers Prev. 2005; 14:1394-1401.

182. Ciresi A, Giordano C. Glucose Metabolism in Children With Growth Hormone Deficiency. Frontiers in Endocrinology. 2018; 9.

183. Thankamony A, Jensen RB, O'Connell SM, Day F, Kirk J, Donaldson M, Ivarsson SA, Soder O, Roche E, Hoey H, Ong KK, Dunger DB, Juul A. Adiposity in Children Born Small for Gestational Age Is Associated With beta-Cell Function, Genetic Variants for Insulin Resistance, and Response to Growth Hormone Treatment. J Clin Endocrinol Metab. 2016; 101:130-141.

184. Lee B, Kim K. Effect of Taekwondo Training on Physical Fitness and Growth Index According to IGF-1 Gene Polymorphism in Children. Korean J Physiol Pharmacol. 2015; 19:341-347.

185. Zheng ZQ, Cao LF, Pei Z, Zhi DJ, Zhao ZH, Xi L, Cheng RQ, Luo FH. Relationship between initial treatment effect of recombinant human growth hormone and exon 3 polymorphism of growth hormone receptor in Chinese children with growth hormone deficiency. International Journal of Clinical and Experimental Medicine. 2015; 8:7965-7970.

186. Moon ES, Kim HS, Sharma V, Park JO, Lee HM, Moon SH, Chong HS. Analysis of Single Nucleotide Polymorphism in Adolescent Idiopathic Scoliosis in Korea: For Personalized Treatment. Yonsei Medical Journal. 2013; 54:500-509.

187. Duijts L, Bakker-Jonges LE, Mook-Kanamori DO, Labout JAM, Hofman A, van Duijn CM, van Dongen JJM, Hooijkaas H, Moll HA, Jaddoe VWV. Variation in the IGF-1 gene is associated with lymphocyte subset counts in neonates: The Generation R Study. Clinical Endocrinology. 2009; 70:53-59.

188. Wang J, Liu HY, Zhang QL. IGF-1 polymorphisms modulate the susceptibility to osteonecrosis of the femoral head among Chinese Han population. Medicine. 2019; 98.

189. Kim H, Park JH, Ku SY, Kim SH, Choi YM, Kim JG. Association between endometriosis and polymorphisms in insulin-like growth factors (IGFs) and IGF-I receptor genes in Korean women. Eur J Obstet Gynecol Reprod Biol. 2011; 156:87-90.

190. Wang Y, Gao C X, Guo TK, Yang RF, Shao FF, Ma WJ, Tian LM. Association of insulin-like growth factor-I receptor and -II receptor gene polymorphisms with osteoporosis in postmenopausal women of Han Chinese. International Journal of Clinical and Experimental Pathology. 2017; 10:2100-2109.

191. van Maldegem AM, Bovee J, Peterse EFP, Hogendoorn PCW, Gelderblom H. Ewing sarcoma: The clinical relevance of the insulin-like growth factor 1 and the poly-ADP-ribose-polymerase pathway. European Journal of Cancer. 2016; 53:171-180.

192. Palsgaard J, Emanuelli B, Winnay JN, Sumara G, Karsenty G, Kahn CR. Cross-talk between insulin and Wnt signaling in preadipocytes: role of Wnt co-receptor low density lipoprotein receptor-related protein-5 (LRP5). J Biol Chem. 2012; 287:12016-12026.

193. Hartleb S, Plockinger U, Stalla GK, Tuschy U, Weber MM, Pfutzner A, Kann P H, German KB, German KPS. Additive effect of GHRd3 and COLIA1 polymorphisms on the GH-substitution dose in GH-deficient adults. Pharmacogenomics. 2011; 12:1653-1661.

194. Zhou GS, Yu JY, Zhang JL. Association between single-nucleotide polymorphisms and haplotype of insulin-like growth factor I and risk of osteoporosis. International Journal of Clinical and Experimental Medicine. 2018; 11:4762-4770.

195. Liu XF, Ma FQ, Zhong YB, Wang J, Li JJ, Shan AJ, Yang C. Association of insulin-like growth factor I gene and vitamin D receptor gene polymorphisms with the risk of osteoporosis in a Chinese population. Biomedical Research-India. 2017; 28:7081-7087.
