# Supplementary material for: A case-control study coupling with meta-analysis elaborates decisive association between IGF-1 rs35767 and osteoporosis in Asian postmenopausal females
Source: Aging (Albany NY). 2023 Jan 3;15(1):134–47. doi: 10.18632/aging.204464 (PMC9876639; doi:10.18632/aging.204464)
Supplement: Supplementary Tables 3 and 4 [file aging-15-204464-s004.pdf]

SUPPLEMENTARY TABLES

Supplementary Table 3. Main characteristics of included studies.

IGF-1 SNP	Study	Race	Patients	Case/Control	HWE
rs35767	Li,2014	Asian	PMOP female	216/220	0.998
	Li,2015	Asian	PMOP female	485/485	0.932
	Wei,2015	Asian	PMOP female	272/272	0.979
	This study	Asian	PMOP female	95/222	0.782
rs2288377	Li,2014	Asian	PMOP female	216/220	<0.001*
	Li,2015	Asian	PMOP female	485/485	<0.001*
	This study	Asian	PMOP female	95/222	0.879
rs5742612	Li,2014	Asian	PMOP female	216/220	<0.001*
	Li,2015	Asian	PMOP female	485/485	<0.001*
	This study	Asian	PMOP female	95/222	0.816

rs35767 Taiwan biobank MAF: 35%; 1000 Genomes MAF: 30%.
rs2288377 Taiwan biobank MAF: 29%; 1000 Genomes MAF: 10%.
rs5742612 Taiwan biobank MAF: 29%; 1000 Genomes MAF: 11%.
*: p-value <0.05.

Supplementary Table 4. *IGF-1* (rs35767, rs2288377, rs5742612) methodological quality of included studies.

	Selection				Comparability		Exposure		
Item/Study	Adequate definition of cases	Representative of the cases	Selection of controls	Definition of controls	Control for age and BMI:	Control for additional factor	Exposure assessment	Same method of ascertainment for all subjects	Non-response rate
Li, 2014	a	a	b	a	a	b	a	a	a
Li, 2015	a	a	b	a	a	a	a	a	a
Wei, 2015	a	a	b	a	a	a	a	a	b

Adequate definition of cases : a: yes, with independent validation. b: yes, eg record linkage or based on self reports. c: no description.
Representativeness of the cases : a: consecutive or obviously representative series of cases; b: potential for selection biases or not stated.
Selection of Controls : a: community controls. b: hospital controls. c: no description.
Definition of Controls : a: no history of disease. b: no description of source.
Control for age and BMI : a: Yes. b: No.
Control for additional factor : a: Yes. b: No.
Exposure assessment : a: secure record (eg surgical records) b: structured interview where blind to case/control status. c: interview not blinded to case/control status. d: written self report or medical record only. e: no description.
Same method of ascertainment for all subjects : a: Yes. b: No.
Non-response rate : a: same rate for both groups. b: non respondents described. c: rate different and no designation.
